# Real-Time Multi-Sensor Joint Fault Diagnosis Method for Permanent Magnet Traction Drive Systems Based on Structural Analysis

**DOI:** 10.3390/s24092878

**Published:** 2024-04-30

**Authors:** Weiwei Gan, Xueming Li, Dong Wei, Rongjun Ding, Kan Liu, Zhiwen Chen

**Affiliations:** 1 College of Mechanical and Vehicle Engineering, Hunan University, Changsha 410082, China; gww@hnu.edu.cn (W.G.); lkan@hnu.edu.cn (K.L.); 2 CRRC Zhuzhou Electric Locomotive Institute Co., Ltd., Zhuzhou 412001, China; lixm10@csrzic.com; 3 School of Automation, Central South University, Changsha 410083, China; zhiwen.chen@csu.edu.cn

**Keywords:** permanent magnet traction drive system, multi-sensors fault, limited monitoring signal, structural model, joint diagnosis

## Abstract

Sensor faults are one of the most common faults that cause performance degradation or functional loss in permanent magnet traction drive systems (PMTDSs). To quickly diagnose faulty sensors, this paper proposes a real-time joint diagnosis method for multi-sensor faults based on structural analysis. Firstly, based on limited monitoring signals on board, a structured model of the system was established using the structural analysis method. The isolation and detectability of faulty sensors were analyzed using the Dulmage–Mendelsohn decomposition method. Secondly, the minimum collision set method was used to calculate the minimum overdetermined equation set, transforming the higher-order system model into multiple related subsystem models, thereby reducing modeling complexity and facilitating system implementation. Next, residual vectors were constructed based on multiple subsystem models, and fault detection and isolation strategies were designed using the correlation between each subsystem model and the relevant sensors. The validation results of the physical testing platform based on online fault data recordings showed that the proposed method could achieve rapid fault detection and the localization of multi-sensor faults in PMTDS and had a good application value.

## 1. Introduction

The traction drive system (TDS) is the only power source of rail transit vehicles, and its energy consumption accounts for about 40~50% of the total energy consumption of rail transit [1]. Permanent magnet traction drive systems (PMTDSs) have become the key development direction of next-generation rail transit traction drive systems [2], due to their advantages of low loss and high efficiency [1]. However, the PMTDS is a high-order complex system with the multi-dimensional coupling of machine electricity–heat magnetism. Hence, it has also become the main source of faults, with increasing service times [3].

**Figure 1:**
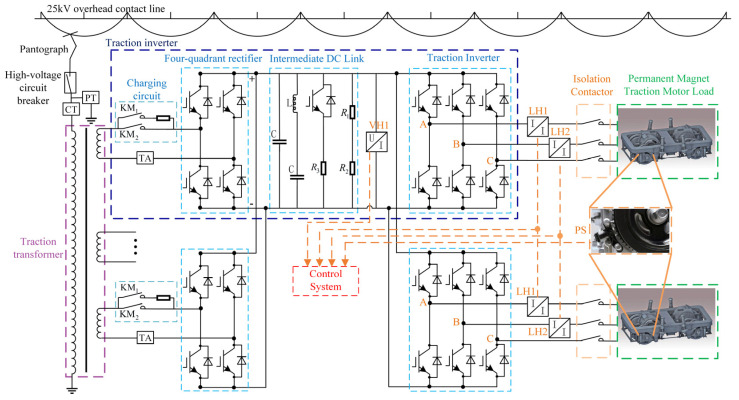
Schematic diagram of the main circuit of a typical PMTDS.

As shown in Figure 1, to realize the high-performance closed-loop control of the permanent magnet traction motor, three kinds of sensors are installed in PMTDS to measure the U- and V-phase currents, the intermediate DC voltage, and the rotor position signal of the motor [4]. Sensors in the traction drive system are susceptible to faults due to mechanical vibration, hot and humid conditions, and strong electromagnetic interferences [5]. Sensor faults can easily lead to control performance degradation and other derivative faults if not detected promptly. Therefore, the study of real-time diagnostic methods of the relevant sensors in PMTDSs has an important engineering application value for improving the reliability and safety of trains.

Fruitful research results have been achieved for the sensor fault diagnosis of PMTDSs [6]. According to the type of diagnostic objects, these methods can be divided into two categories: single-sensor fault diagnosis and joint fault diagnosis of multiple types of sensors. Regarding single-sensor faults, current sensor faults are diagnosed using different methods, including current estimation [7], a phase-locked loop-based current reconstruction technique [8], an adaptive observer [9], and a sliding-mode observer [10]. In terms of motor speed or position sensors, diagnostic methods have been proposed based on q-axis current fault characteristics’ analysis [11], look-up table [12], Kalman filter [13], and the speed estimation model [14].

Compared to single-type sensor fault diagnosis, multi-sensor joint fault diagnosis is relatively less researched, because a more complex multivariate estimation model is required [15]. Multiple independent observers-based methods [1,16], the signal processing-based method [17], and the data-driven method [18] have been proposed to achieve the joint diagnosis of intermediate DC voltages, motor currents, and speed sensor faults. However, the above methods have the disadvantages of their computational burden, reliance on the analytic redundancy of three-phase currents, and poor generalization ability, which are not implementation-friendly. The recently proposed convolutional vector fusion network [19], the semi-supervised matrixed graph-embedding machine [20], and the non-parallel bounded-support matrix machine [21] suffer from the interpretability issue. In this paper, the model-based multi-sensor fault joint diagnosis of PMTDSs is investigated to improve its engineering level further.

Structural analysis is a model-based method [22] that decomposes a complex system into several subsystems. Following this step, the diagnosis of the related faults in a system by mining the set of analytically redundant relations in the system is carried out [23,24]. Zhang J. et al. studied the joint diagnosis problem for eight sensor faults in the permanent magnet drive system of electric vehicles, including inverter output three-phase voltage, motor output three-phase current, a motor position sensor, and a vehicle speed sensor [23]. Ebrahimi S. H. et al. realized the joint diagnosis of the position sensor and the motor [24]. The diagnosis of 10 sensor signals was investigated, including inverter input DC voltage, inverter output three-phase voltage, motor three-phase current, motor speed, motor position, and load torque, based on the inverter output three-phase voltage, the motor output three-phase current, and the motor position sensor signals in a permanent magnet drive system [25]. The above multi-sensor joint diagnosis methods based on structural analysis [23,24,25] have added many redundant sensors (e.g., hardware redundancy among three-phase voltage sensors, redundancy between motor speed and position sensors, etc.) to simplify the redundancy relationship of sequence residual analysis. In contrast, such redundant sensors have not been arranged in the actual system considering the cost and reliability factors. Therefore, the above diagnostic methods have some limitations in real-time fault diagnosis tasks.

This paper investigates the real-time multi-sensor joint fault diagnosis of PMTDSs based on the structural analysis method by making full use of the intermediate DC voltage, the motor currents of phases A and B, and the rotor position information collected from the closed-loop control of the PMTDS. The main contributions are as follows:

1. A real-time joint diagnosis method for the faults of the intermediate DC voltage sensor, the A- and B-phase current sensors, and the position sensor in PMTDSs is proposed;
2. The detectability and isolability of each sensor fault with limited sampling signals are presented, and residuals are generated by the analytic redundancy relationship. Different combinations of residuals are used to realize the fast and effective isolation of all the sensors.
3. A diagnostic algorithm test verification method based on data recording to reproduce real fault scenarios is proposed, and a relevant test platform is built to verify the effectiveness of the proposed diagnostic method.

## 2. Basis for the Decomposable Diagnosis of Sensor Faults in a System

### 2.1. Mathematical and Structured Modeling of PMTDSs

The main circuit of a typical PMTDS for locomotives and rolling stock is shown in Figure 1, and it mainly consists of three parts: the traction transformer, the traction converter (including the charging circuit, the four-quadrant rectifier, the intermediate DC link, the traction inverter, etc.), and the PM traction motor. To realize the real-time closed-loop control of the permanent magnet traction motor, the related sensors are defined as shown in Table 1.

According to the circuit principle, the mathematical model of the permanent magnet traction drive system can be obtained as shown in Equation (1), where *e*_1_–*e*_21_ represent subequations, and the meaning of each variable is shown in Table 2.

(1) $$\begin{array}{l} e_{1}:di_{d}=\frac{1}{L_{d}}(u_{d}-R_{s}i_{d}+\omega_{e}L_{q}i_{q})\\ e_{2}:di_{q}=\frac{1}{L_{q}}(u_{q}-R_{s}i_{q}-\omega_{e}L_{d}i_{d}-\omega_{e}\psi_{f})\\ e_{3}:d\theta_{e}=\omega_{e}\\ e_{4}:u_{d}=u_{\alpha }\cos\theta_{e}+u_{\beta }\sin\theta_{e}\\ e_{5}:u_{q}=-u_{\alpha }\sin\theta_{e}+u_{\beta }\cos\theta_{e}\\ e_{6}:u_{\alpha }=\frac{2}{3}(u_{a}-0.5u_{b}-0.5u_{c})\\ e_{7}:u_{\beta }=\frac{1}{\sqrt{3}}(u_{b}-u_{c})\\ e_{8}:u_{a}=\frac{U_{dc}}{3}(2S_{a}-S_{b}-S_{c})\\ e_{9}:u_{b}=\frac{U_{dc}}{3}(2S_{b}-S_{a}-S_{c})\\ e_{10}:u_{c}=\frac{U_{dc}}{3}(2S_{c}-S_{a}-S_{b})\\ e_{11}:i_{d}=i_{\alpha }\cos\theta_{e}+i_{\beta }\sin\theta_{e}\\ e_{12}:i_{q}=-i_{\alpha }\sin\theta_{e}+i_{\beta }\cos\theta_{e}\\ e_{13}:i_{\alpha }=i_{a}\\ e_{14}:i_{\beta }=\frac{1}{\sqrt{3}}i_{a}+\frac{2}{\sqrt{3}}i_{b}\\ e_{15}:y_{Udc}=U_{dc}+f_{Udc}\\ e_{16}:y_{Ia}=i_{a}+f_{Ia}\\ e_{17}:y_{Ib}=i_{b}+f_{Ib}\\ e_{18}:y_{\theta n}=\frac{1}{n_{p}}\theta_{e}+f_{\theta n}\\ e_{19}:di_{d}=\frac{d}{dt}i_{d}\\ e_{20}:di_{q}=\frac{d}{dt}i_{q}\\ e_{21}:d\theta_{e}=\frac{d}{dt}\theta_{e} \end{array}$$

**Figure 2:**
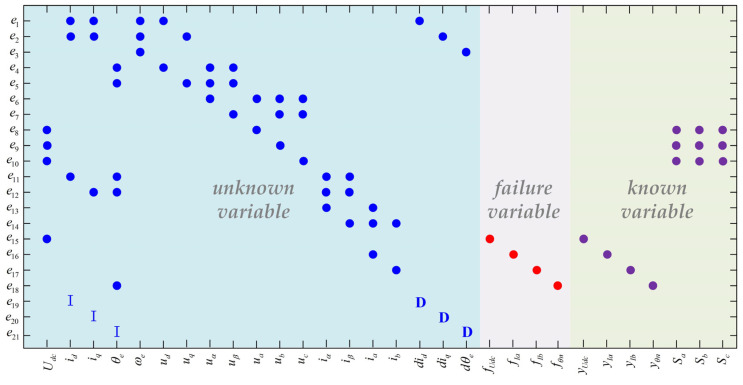
Structural model of traction converter.

The structured model can describe the relationship of the variables in the system’s mathematical model through the association matrix’s structure and clearly express the relationship between the equations and the variables, such as in Figure 2, which shows the structured model of the traction system. The structured model divides the variables into three categories as follows:

unknown variable: {*U_dc_*, *i_d_*, *i_q_*, *θ_e_*, *ω_e_*, *u_d_*, *u_q_*, *u_α_*, *u_β_*, *u_a_*, *u_b_*, *u_c_*, *i_α_*, *i_β_*, *θ_n_*}

failure variable: {*f_Udc_*, *f_Ia_*, *f_Ib_*, *f_θn_*}

known variable: {*y_Udc_*, *y_Ia_*, *y_Ib_*, *y_θn_*, *S_a_*, *S_b_*, *S_c_*}

In Figure 2, D denotes the differential variable relationship, and I denotes the integral variable relationship. Subequations *e*_15_~*e*_18_ describe the relationship between the sensor measurements of the known variables’ intermediate DC voltage, the stator phase-A and -B currents, and the rotor’s position and the corresponding fault quantities and true values of each measurement.

### 2.2. Detectability and Isolation of Sensor Faults in System

The structural analysis method is concerned with the structurally overdetermined part. The fact that the number of equations in a structural model is greater than the number of unknown variables implies that it is structurally analytically redundant, and this redundancy information can be utilized to generate residual values for fault diagnosis.

**Figure 3:**
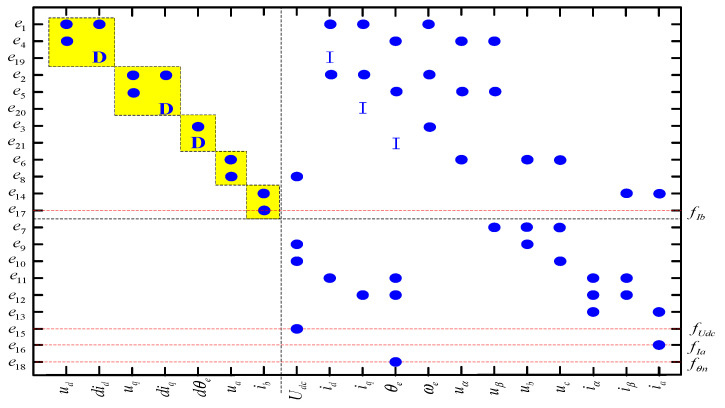
DM decomposition results for the structural model of a PMTDS.

A Dulmage–Mendelsohn (DM) decomposition [21] of the structured model of PMTDS (1) was performed, which was used for deriving the redundancy relationship of the system’s structural model. The canonical decomposition of its overdetermined part is shown in Figure 3, from which it can be seen that all the defined faults appear in the overdetermined part. Therefore, all the sensor faults of the system listed in Table 2 are detectable.

**Figure 4:**
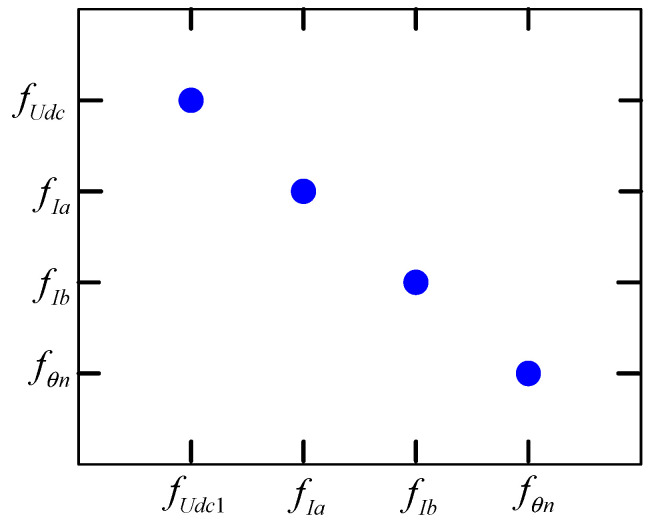
Fault isolation matrix.

Fault isolability consists of recognizing and isolating a fault from other faults when the new fault occurs. From the literature [21], in a system model, a fault can be isolated from the other faults if the fault satisfies the relationship in Equation (2).

(2) $$e_{f_{i}}\in {\left(M/\{e_{f_{j}}\}\right)}^{+}$$

where $e_{f_{i}}$ and $e_{f_{j}}$ are the subequations containing the faults, and $M/{\left\{e_{f_{j}}\right\}}^{+}$ is the overdetermined part of the structure after eliminating the equations. According to the definition of fault isolability, the fault isolation matrix can be obtained as shown in Figure 4. The faults *f_U__dc_*, *f_I__a_*, *f_I__b_*, and *f_θn_* are only correlated with themselves, indicating that the faults of the DC bus voltage sensor, the two-phase current sensors of A and B, and the rotor position sensor are isolatable.

### 2.3. Calculation of Minimum Set of Super-Deterministic Equations

In order to generate sequence residuals for fault diagnosis, it was first necessary to determine the structural minimum set of overdetermined equations, MSOs, i.e., the smallest number of equations for which maximum isolability can be achieved. The MSOs are subsets of the set of equations with analytic redundancy. In this paper, based on all the MSOs of the structured model, the minimum touch set approach [26] was used to obtain four structural minimal super-determined sets of equations, MSOs, as shown in Table 3.

According to the structural analysis method, the above structural minimum set of super-deterministic equations MSOs could be used to generate four independent residuals, and the joint use of these MSOs isolated all the faults mentioned above, as shown in Table 4.

In Table 4, the symbol “X” indicates that the fault is detectable, and a blank space indicates that the fault is not detectable. For example, the over-determined set of equations MSO_1_ was able to detect three faults ($f_{Udc}$, $f_{Ia}$, and $f_{\theta n}$) but not the other faults.

## 3. Design of Multi-Sensor Fault Joint Diagnosis Algorithm

### 3.1. Sequence Residual Design

#### 3.1.1. Residual *R*_1_ and *R*_4_

As it can be seen from Table 4, the two overdetermined equation sets MSO_1_ and MSO_4_ both contained an intermediate voltage sensor, a position sensor signal, and a phase current sensor signal. Therefore, we could estimate a phase current by establishing a current observer and establish the corresponding residual error using the current estimation error.

According to the state equation of a permanent magnet synchronous motor in the rotating coordinate system of the d- and q-axis rotor, a state observer was designed to estimate the state and the output [27], and the observation errors of the A-phase current and B-phase current were used to constitute the correction, respectively. The observer state space equation corresponding to the residual *R*_1_ and *R*_4_ can be expressed as follows:

(3) $$\left\{\begin{array}{l} \frac{d{\hat{x}}_{1}}{dt}=A{\hat{x}}_{1}+Bu+B_{d}l+K_{1}(y_{Ia}-{\hat{y}}_{1})\\ {\hat{y}}_{1}=C_{1}x_{1} \end{array}\right.$$

(4) $$\left\{\begin{array}{l} \frac{d{\hat{x}}_{2}}{dt}=A{\hat{x}}_{2}+Bu+B_{d}l+K_{2}(y_{Ib}-{\hat{y}}_{2})\\ {\hat{y}}_{2}=C_{2}x_{2} \end{array}\right.$$

where

$$A=[\begin{array}{cc} -R_{s}/L_{d} & \omega_{e}L_{q}/L_{d}\\ -\omega_{e}L_{d}/L_{q} & -R_{s}/L_{q} \end{array}],\;B=[\begin{array}{cc} 1/L_{d} & 0\\ 0 & 1/L_{q} \end{array}],$$

$$B_{d}=[\begin{array}{c} -\psi_{f}/L_{q}\\ 0 \end{array}],x=[\begin{array}{c} i_{d}\\ i_{q} \end{array}],u=[\begin{array}{c} u_{d}\\ u_{q} \end{array}],l=\omega_{e}$$

$$C_{1}=[\begin{array}{cc} \cos\theta_{e} & -\sin\theta_{e} \end{array}],C_{2}=[\begin{array}{cc} \sqrt{3}/2\sin\theta_{e}-1/2\cos\theta_{e} & 1/2\sin\theta_{e}+\sqrt{3}/2\cos\theta_{e} \end{array}]$$

*K*_1_ and *K*_2_ are observer gain matrices whose values are set according to the requirements of stability, fault sensitivity, and robustness.

Based on the observer described in Equations (3) and (4), residuals *R*_1_ and *R*_4_ can be designed as shown in Equations (5) and (6), respectively.

(5) $$R_{1}(k)=y_{Ia}(k)-{\hat{y}}_{1}(k)$$

(6) $$R_{4}(k)=y_{Ib}(k)-{\hat{y}}_{2}(k)$$

#### 3.1.2. Residual *R*_2_

The equation set MSO_2_ consists of 20 equations that generate residual *R*_2_ to detect faults. From correlation Equation (1), the following can be obtained:

(7) $$\begin{array}{l} {\hat{u}}_{q}\cdot [(2S_{a}-S_{b}-S_{c})\cdot \cos(n_{p}\cdot y_{\theta n})+\sqrt{3}(S_{b}-S_{c})\cdot \sin(n_{p}\cdot y_{\theta n})]\\ ={\hat{u}}_{d}\cdot [-(2S_{a}-S_{b}-S_{c})\cdot \sin(n_{p}\cdot y_{\theta n})+\sqrt{3}(S_{b}-S_{c})\cdot \cos(n_{p}\cdot y_{\theta n})] \end{array}$$

where the expressions of ${\hat{u}}_{d}$ and ${\hat{u}}_{q}$ are the estimated voltage values of the output d-axis and q-axis of the inverter, respectively, and the expression is shown in Equation (8).

(8) $$\left\{\begin{array}{l} {\hat{u}}_{d}=L_{d}\frac{di_{d}}{dt}+R_{s}i_{d}-n_{p}\cdot L_{q}\cdot i_{q}\cdot \frac{d\theta_{n}}{dt}\\ {\hat{u}}_{q}=L_{q}\frac{di_{q}}{dt}+R_{s}i_{q}+n_{p}\cdot (L_{d}\cdot i_{d}+\psi_{f})\cdot \frac{d\theta_{n}}{dt} \end{array}\right.$$

The first-order backward difference is used to discretize Equation (8), and the residual *R*_2_(*k*) can be obtained.

(9) $$\begin{array}{l} R_{2}(k)={\hat{u}}_{q}(k)\cdot [(2S_{a}-S_{b}-S_{c})\cdot \cos(n_{p}\cdot y_{\theta n})+\\ \;\;\;\;\sqrt{3}(S_{b}-S_{c})\cdot \sin(n_{p}\cdot y_{\theta n})]+\\ \;\;\;\;{\hat{u}}_{d}(k)\cdot [(2S_{a}-S_{b}-S_{c})\cdot \sin(n_{p}\cdot y_{\theta n})-\\ \;\;\;\;\sqrt{3}(S_{b}-S_{c})\cdot \cos(n_{p}\cdot y_{\theta n})] \end{array}$$

#### 3.1.3. Residual *R*_3_

As it can be seen from Table 4, MOS3-related parties included a related signal in the equations, so we estimated the rotor speed and rotor position information by establishing an MRAS based on Popov’s super-stability theorem and feeding it back to the current observer [27] and by establishing a residual error by motor current estimation. Taking the phase-A current estimation error as residual *R*_3_, we could obtain

(10) $$R_{3}(k)=y_{Ia}(k)-({\hat{i}}_{d}(k)\cdot \cos{\hat{\theta }}_{e}(k)-{\hat{i}}_{q}(k)\cdot \sin{\hat{\theta }}_{e}(k))$$

### 3.2. Fault Detection and Decision

#### 3.2.1. Periodic Adaptive Fault Detection Strategy

Due to the influence of nonlinear factors such as intermediate DC voltage fluctuation, dead time, and tube voltage drop, the output voltage of the inverter reconstructed by the IGBT pulse state and intermediate voltage have a certain deviation from the actual voltage, and the residual is shown as high-frequency harmonics. In addition, the residual signal characteristics have a strong correlation with the inverter output voltage and current frequency. Therefore, based on the actual output voltage and current frequency, this paper uses a periodic adaptive sliding window to construct periodic detection statistics for fault detection, which can avoid the influence of different speeds on residual calculation.

It is assumed that, in the normal operation, the residual is satisfied as *R*~*N* (*μ*_0_, $\sigma_{0}^{2}$), where $\mu_{0}$ = 0 is the mean of normal residuals, and $\sigma_{0}^{2}$ is related to the measurement noise and harmonics of residuals that can be obtained by learning a large number of historical data from the site under normal working conditions. Let $\tilde{R}=\{R^{1},R^{2},\cdots ,R^{N}\}$ be the periodic sampling value of *R_i_*.

The detection statistics are defined as follows:

(11) $$T^{2}=\frac{\sum_{i=1}^{N}{\left(R^{i}\right)}^{2}}{\sigma_{0}^{2}}$$

where $T^{2}$ satisfies the standard Chi-square distribution, denoted as $\chi^{2}$, with *N* degrees of freedom, and $p(T^{2}>\chi_{\alpha }^{2}|H_{0})=\alpha$. $p(T^{2}>\chi_{\alpha }^{2}|H_{0})=\alpha$ denotes the probability that the detection statistic $T^{2}$ is larger than $\chi_{\alpha }^{2}$ under the fault-free assumption H_0_.

This method was adopted for fault detection, and the threshold value was obtained by an approximate Chi-square distribution, that is [28,29]

(12) $$T_{\alpha }=\chi_{\alpha }^{2}(N-1)$$

where $T_{\alpha }$ represents the detection threshold, $\chi_{\alpha }^{2}(N-1)$ represents the Chi-square distribution of *N* − 1 degrees of freedom, and α represents the confidence level, which is generally understood as the probability of allowing false detection. $N=f_{s}/f_{0}$ is the number of sampled data points in the sliding window, the signal sampling rate is $f_{s}$, and the inverter output current fundamental frequency is $f_{0}$.

The corresponding fault detection decision logic can be expressed as

(13) $$F=\left\{\begin{array}{l} 1,\;T^{2}>T_{\alpha }\;\\ 0,\;T^{2}\leq T_{\alpha } \end{array}\right.$$

where *F* represents the detected fault status flag, 1 is the fault state, and 0 is the normal state.

#### 3.2.2. Fault Decision Making

Assuming that the fault detection results corresponding to the four residuals are $F_{Ri},i=1,\cdots ,4$, combined with the fault feature matrix in Table 4, the effective detection and isolation of each fault can be achieved based on the diagnosis rules shown in Table 5.

**Figure 5:**
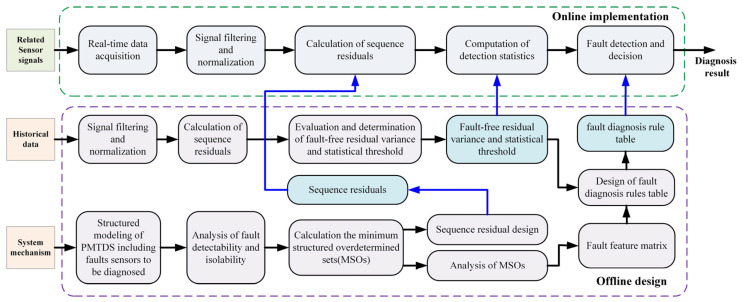
Basic framework of the proposed method.

The basic framework of the proposed diagnostic algorithm is shown in Figure 5, and the entire algorithm is divided into two parts: offline design and online implementation. The offline design part mainly completes the design of the residual and sets corresponding thresholds. In the online implementation part, the system collects real-time sensor and system status information, filters and normalizes the signal, and calculates the residual and the related detection statistics in real time based on the offline-designed residual expression. Combined with the offline-designed threshold parameters and the fault feature matrix, it can realize the effective detection and diagnosis decision of different sensor faults.

## 4. Testing and Verification

### 4.1. Diagnostic Objects and Test Platforms

Based on the PMTDS of a certain train (the main relevant parameters are shown in Table 6), this model simulates the faults of various sensors through the semi-physical simulation test platform and records the relevant data, and then introduces them to the physical test verification platform based on the online recording of fault data to test and verify the proposed algorithm.

**Figure 6:**
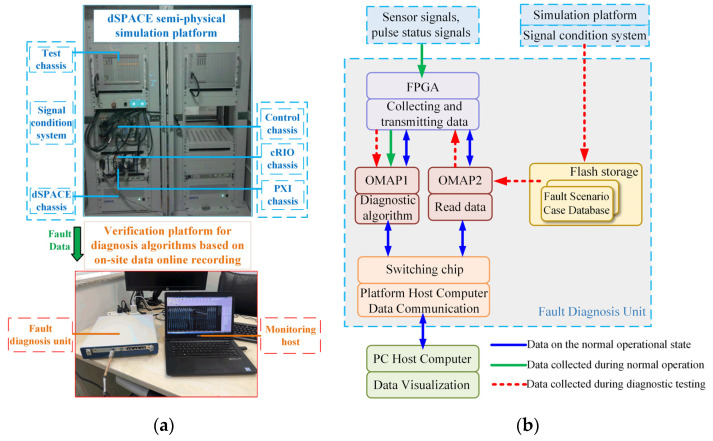
Physical testing and verification platform of the diagnosis algorithm based on online fault data recording: (**a**) the physical diagram; and (**b**) the hardware architecture of the fault diagnosis unit.

The whole experimental system is divided into two parts: the dSPACE semi-physical simulation platform and the diagnosis algorithm verification platform based on online fault data recording. The physical diagram is shown in Figure 6a. Among them, the semi-physical simulation platform is mainly composed of a real controller (control chassis), a real-time simulator (dSPACE chassis), and a signal-conditioning system. The semi-physical simulation platform is used to simulate various sensor faults on site and generate and save the related fault data records. Then, the Flash memory is transmitted to the fault diagnosis unit by the host computer and is monitored for the diagnostic algorithm test. The verification platform of the diagnosis algorithm consists of a fault diagnosis unit and a monitoring host unit. The hardware architecture of the fault diagnosis unit is shown in Figure 6b, and it is mainly composed of two OMAPL138 chips, an FPGA chip, and a Flash memory. The real-time diagnosis algorithm is implemented in the OMAP1 chip of the fault diagnosis unit. When the real-time diagnosis algorithm is tested, the OMAP2 chip reads the fault data file from the Flash memory into the memory and sends it to OMAP1 via FPGA to complete the true representation of the on-site fault scene.

**Figure 7:**
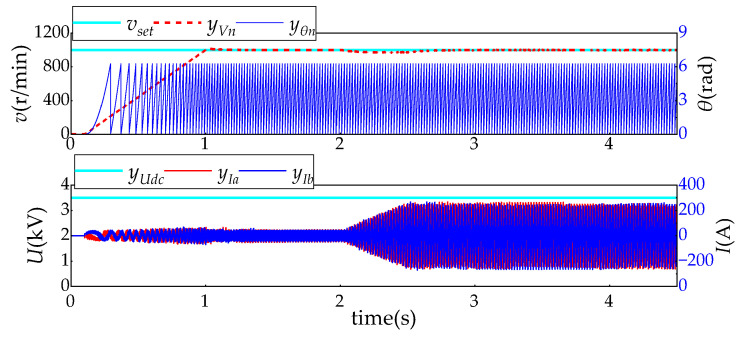
System response curve under normal working conditions.

The main parameters of the traction drive system are shown in Table 6. When *t* = 0.1 s, the traction inverter is started, the set speed is linearly increased from 0 to 1000 r/min, and the actual speed is sampled in real time for closed-loop constant speed control. When *t* = 2 s, 2000 Nm torque is loaded, and then different types of sensor fault signals are injected when *t* = 4 s, and the relevant fault data waveforms are collected for real-time diagnosis algorithm testing. The system control response curve under normal working conditions is shown in Figure 7.

### 4.2. Experiment Results

**Figure 8:**
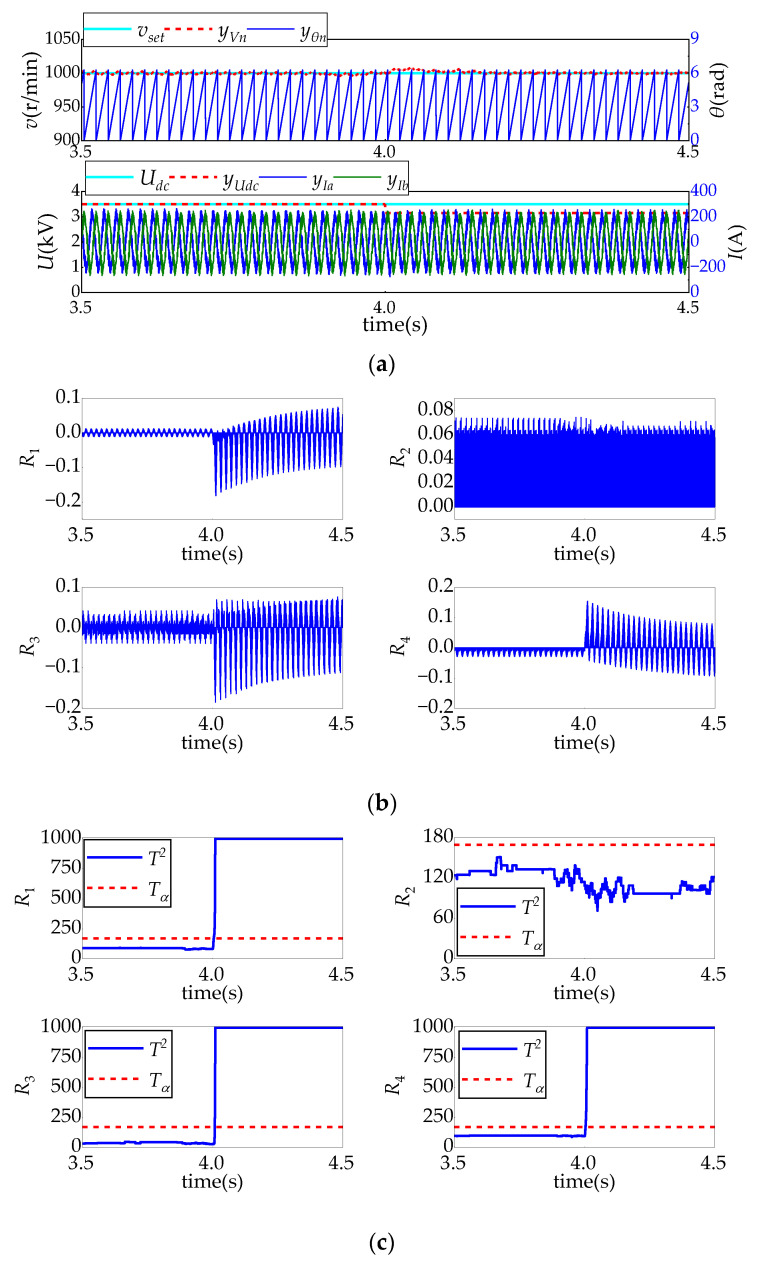
Intermediate DC voltage sensor fault test result: (**a**) relevant sensor sampling signal and system control response; (**b**) residual changes; and (**c**) detection of changes.

**Figure 9:**
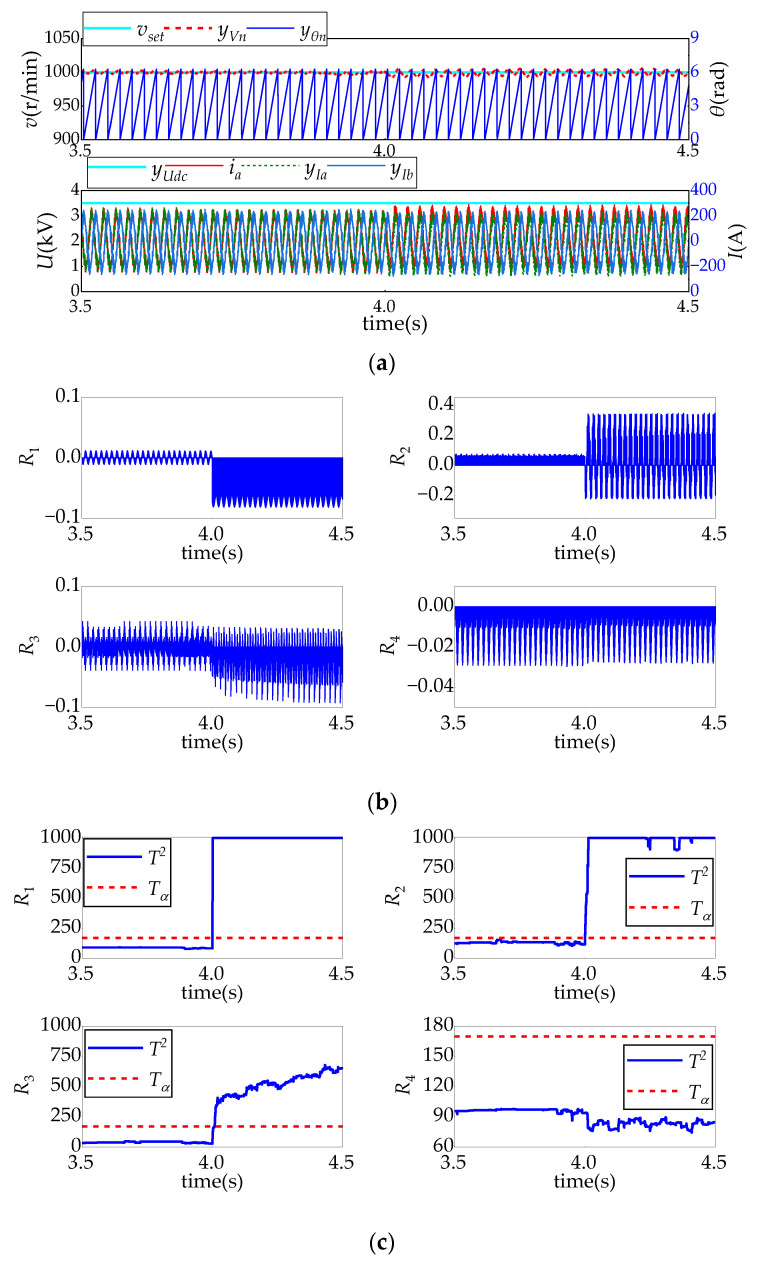
Motor A-phase current sensor fault test results: (**a**) relevant sensor sampling signal and system control response; (**b**) residual changes; and (**c**) detect changes in statistics.

**Figure 10:**
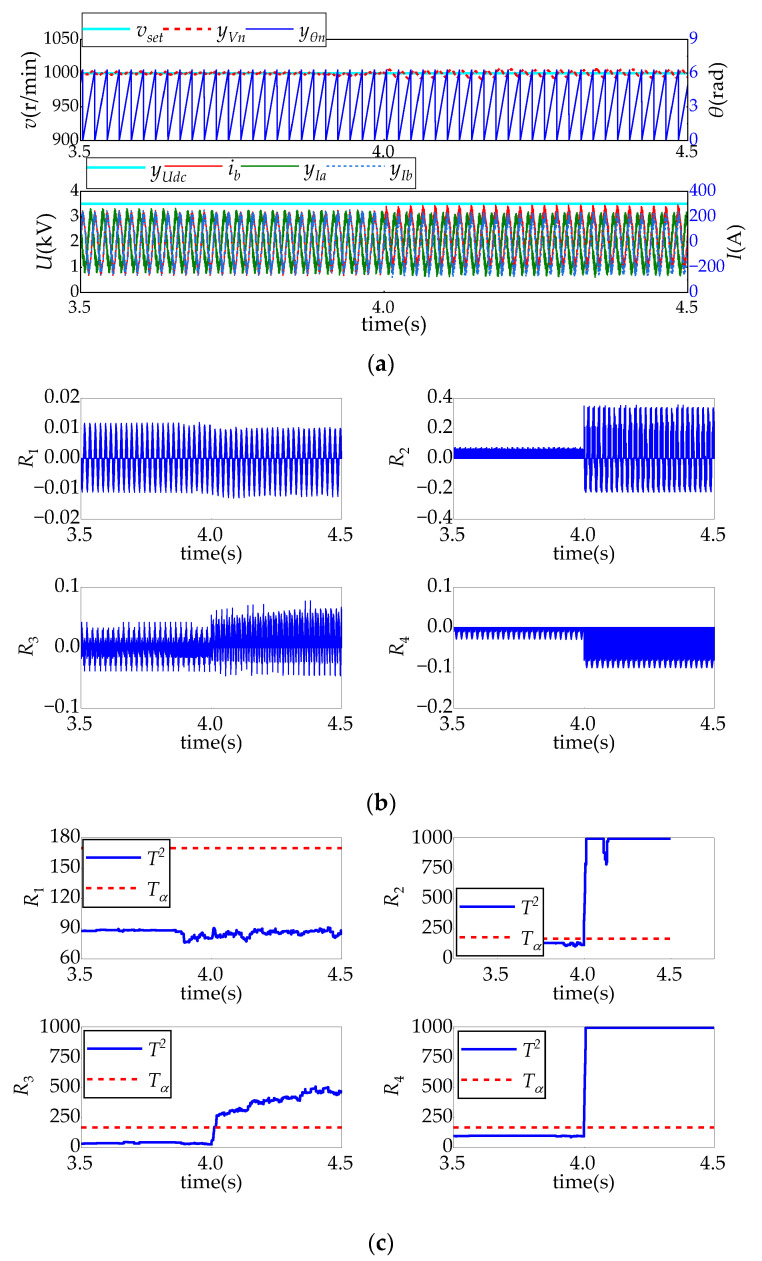
Motor B-phase current sensor fault test results: (**a**) relevant sensor sampling signal and system control response; (**b**) residual changes; and (**c**) detection of changes.

**Figure 11:**
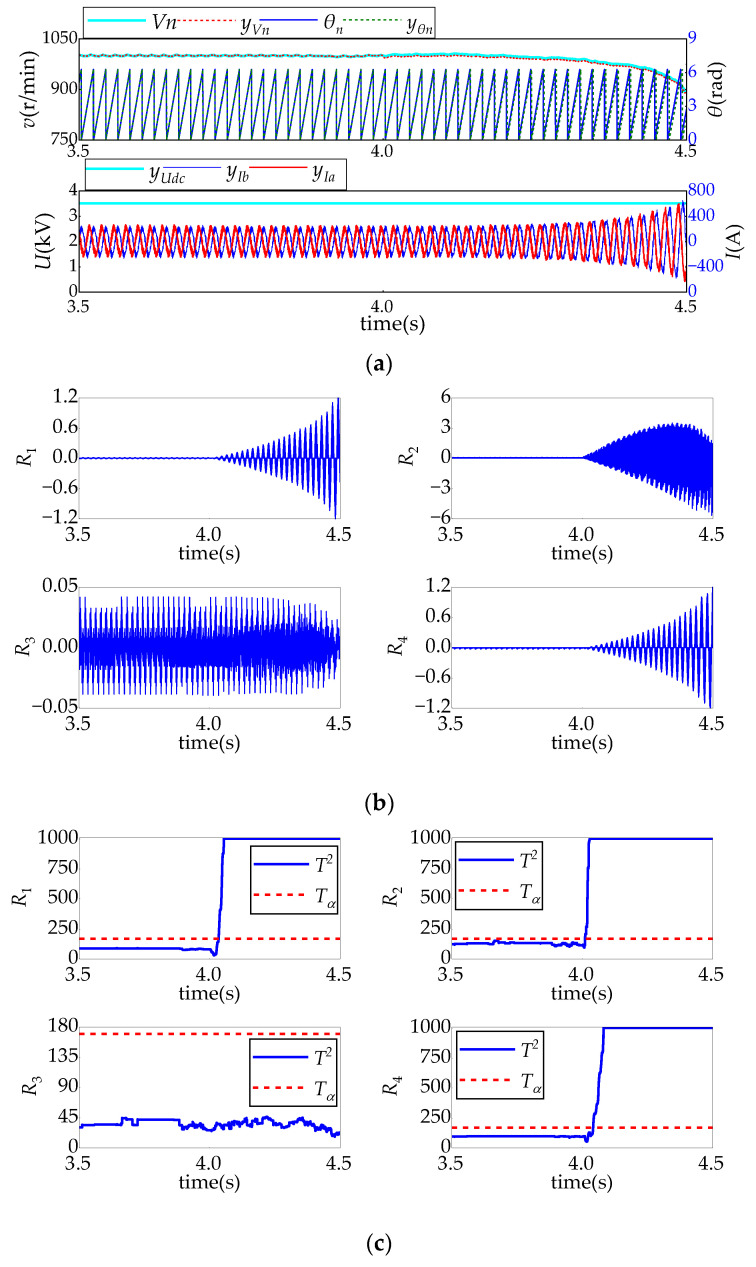
Motor position sensor fault test results: (**a**) relevant sensor sampling signal and system control response; (**b**) residual changes; and (**c**) detection of changes.

Figure 8, Figure 9, Figure 10 and Figure 11 show the system’s response and the real-time fault diagnosis results under different sensor fault conditions. In these figures, in order to prevent data overflow without affecting the diagnosis results, the maximum value of detection statistics in the diagnosis algorithm is limited to 1000.

Figure 8 shows the diagnostic test results when the simulated intermediate voltage sensor has a 10% deviation fault after *t* = 4 s. As it can be seen from Figure 8a, due to the closed-loop control of the system, the motor speed signal fluctuates slightly after the fault and becomes stable rapidly, while the motor current does not fluctuate significantly. The reason is that the 10% negative deviation measured mid-voltage will lead to an increase in the duration times of the applied voltage vectors in the space vector pulse width modulation (SVPWM), thus resulting in larger reference voltage and torque. Accordingly, the speed will increase slightly and then decrease to a steady value due to the regulation of the speed-loop controller.

However, it is easy to see from Figure 8b that residuals *R*_1_, *R*_3_, and *R*_4_ all show significant changes, and their corresponding detection statistics all exceed their detection thresholds, meeting the fault diagnosis rules of the intermediate voltage sensors in Table 5.

Figure 9 and Figure 10 are the fault test results simulating the −50 A deviation fault of the A- and B-phase current sensors of the motor, respectively, when *t* = 4 s. It can be seen from the corresponding subfigure (a) that, after the motor current sensor fails, the system speed fluctuates greatly. Due to the closed-loop regulation of the system, the fault-phase current signal is adjusted to the amplitude of the normal-phase current, increasing the amplitude of the true fault-phase current compared to that of the normal-phase current, which is about the size of the deviated fault current. If the sensor fault is not diagnosed in time, the risk of system overcurrent increases. It can be seen from the corresponding Figure 9b and Figure 10b that, after the fault, the corresponding residuals and detection statistics change laws are consistent with the faults of the A-phase and B-phase current sensors of the motor in Table 5, and the system can normally diagnose the faults of the A-phase and B-phase current sensors of the motor.

An abnormal motor position signal can easily lead to system control malfunction. Figure 11 shows the diagnostic results of a 0.5% deviation fault of the motor speed sensor signal when *t* = 4 s is simulated. As it can be seen from Figure 11a, a deviation in the speed-sampling signal will cause the deviation between the position signal and the real position signal to gradually increase, the constant speed control of the motor will gradually fail, and the motor current will rapidly diverge. It can be seen from Figure 11b that, with the control malfunction, the amplitudes of residuals *R*_1_, *R*_2_, and *R*_4_ correspondingly increase rapidly, their corresponding detection statistics rapidly exceed the detection threshold, and the system can correctly diagnose the fault of the motor position sensor.

Regarding the diagnosis of sensor faults in permanent magnet traction transmission systems, there are currently three main methods: model-based, signal-based, and data-driven methods. Model-based methods have a small computational complexity and a fast diagnostic speed, but they require a high accuracy of the model and are sensitive to system parameter perturbations, resulting in the low robustness of their diagnostic strategy. The method based on current signals does not require system parameters, but noise, load disturbances, and different load conditions may bring uncertainty to its diagnosis, resulting in a high rate of false alarm. The data-driven diagnostic method does not require an accurate system-analytical model when dealing with fault diagnosis problems in complex systems, but it has problems such as a large computational complexity, unclear physical concepts, and generally slow response speeds to faults. Hence, according to the experimental results, the proposed structured model-based joint diagnosis method in this paper overcomes the shortcomings of the above methods, and the faults of the intermediate DC voltage sensor, the A- and B-phase current sensors, and the position sensor in PMTDSs can be correctly diagnosed in real time.

## 5. Conclusions

Based on the typical sensor layout of PMTDSs in practical engineering, the diagnosability of each sensor fault was hereby demonstrated, and a real-time joint diagnosis method for the multi-sensor fault of permanent magnet traction systems was proposed. This method is based on the structured model of permanent magnet traction systems, including sensor faults, and takes full account of the correlation between the sensor signals to establish the residual. It has the advantages of a clear physical concept, simple implementation, and a small amount of calculation and has good engineering application prospects.

However, due to the use of the IGBT status, the motor parameters, and other information in structured modeling, when an IGBT fault occurs in the system, the change in the motor parameters will affect the diagnosis results. Therefore, our future research will add IGBT faults to the model and increase the real-time identification strategy of the motor parameters to improve the adaptability of the proposed method.

## Figures and Tables

**Table 1:** Permanent magnet traction transmission system-related sensors.

Sensor Code	Definition
VH1	Intermediate DC voltage sensor
LH1	Motor A-phase current sensor
LH2	Motor B-phase current sensor
PS	Position sensor

**Table 2:** Meanings of variables.

Symbol	Meaning
*U_dc_*	Intermediate DC voltage
*i_d_*	d-axis current of the motor
*i_q_*	q-axis current of the motor
*θ_e_*	Motor rotor angular position
*ω_e_*	Motor rotor angular speed
*u_d_*	Inverter output d-axis voltage
*u_q_*	Inverter output q-axis voltage
*u_α_*	The inverter outputs the α-axis voltage
*u_β_*	The inverter outputs the β-axis voltage
*u_a_*	The inverter outputs the A-phase voltage
*u_b_*	The inverter outputs the B-phase voltage
*u_c_*	The inverter outputs the C-phase voltage
*i_α_*	Motor α-axis current
*i_β_*	Motor β-axis current
*di_d_*	Differential of d-axis current of motor
*di_q_*	Differential of q-axis current of motor
*dθ_e_*	Differential angle position of motor rotor
*S_a_*, *S_b_*, *S_c_*	Inverter pulse control signal
*y_Udc_*	Sampling value of the intermediate DC voltage sensor
*y_Ia_*	Motor phase-A current sensor sampling value
*y_Ib_*	Motor phase-B current sensor sampling value
*y_θn_*	Motor rotor position sensor sampling value
*f_Udc_*	The intermediate DC voltage sensor fault
*f_Ia_*	The A-phase current sensor of the motor fault
*f_Ib_*	The B-phase current sensor of the motor fault
*f_θn_*	The motor rotor position sensor fault
*R_s_*	Stator resistance
*L_d_*	Motor d-axis inductance
*L_q_*	Motor q-axis inductance
*ψ_f_*	Rotor permanent magnet linkage
*n_p_*	Number of motor poles

**Table 3:** Minimum set of overdetermined equations.

Equations Set	Including Equations
MSO_1_	*e*_1_~*e*_13_, *e*_15_~*e*_16_, *e*_18_~*e*_21_
MSO_2_	*e*_1_~*e*_14_, *e*_16_~*e*_21_
MSO_3_	*e*_1_~*e*_17_, *e*_19_~*e*_21_
MSO_4_	*e*_1_~*e*_15_, *e*_17_~*e*_21_

**Table 4:** Attributes of the selected MSO sets.

Equations	$f_{Udc}$	$f_{Ia}$	$f_{Ib}$	$f_{\theta n}$
MSO_1_	X	X		X
MSO_2_		X	X	X
MSO_3_	X	X	X	
MSO_4_	X		X	X

**Table 5:** Fault diagnosis rule table.

Rules	Precondition				Conclusion
Code	*F_R_*_1_	*F_R_*_2_	*F_R_*_3_	*F_R_*_4_	
1	1	0	1	1	*f_Udc_* = 1
2	1	1	1	0	*f_Ia_* = 1
3	0	1	1	1	*f_Ib_* = 1
4	1		0	1	*f_θn_* = 1

**Table 6:** Description of the main parameters of PMTDSs.

Parameters	Value
Rated intermediate voltage of the converter/V	3500
Rated output voltage/V	2517
Rated torque/(Nm)	5994
Rated speed/(r·min^−1^)	2274
Rated current of permanent magnet motor (rms)/A	351
Maximum current of permanent magnet motor (rms)/A	490
Rated power/kW	1430
Stator resistance/Ω	0.03
Direct axis inductance/mH	2.97
Quadrature axis inductance/mH	8.49
Permanent magnet flux linkage/Wb	1.92
Number of motor poles	3

## Data Availability

No new data were created or analyzed in this study. Data sharing is not applicable to this article.
